# Supplementary material for: Is amalgam removal in patients with medically unexplained physical symptoms cost-effective? A prospective cohort and decision modelling study in Norway
Source: PLoS One. 2022 Apr 29;17(4):e0267236. doi: 10.1371/journal.pone.0267236 (PMC9053791; doi:10.1371/journal.pone.0267236)
Supplement: S1 Table — (DOCX) [file pone.0267236.s001.docx]

# **Supporting information**

**S1 Table.** **Annual per person costs in the Amalgam and MUPS cohort (in NOK) at baseline and follow-up**

|  | Amalgam (n=32) | | | | MUPS (n=28) | | | |
| --- | --- | --- | --- | --- | --- | --- | --- | --- |
| Cost components | Baseline | | Follow-up | | Baseline | | Follow-up | |
|  | Mean | SD | Mean | SD | Mean | SD | Mean | SD |
| A. Cost of amalgam removal (once-off) | |  |  |  |  |  |  |  |
| Intervention costs |  |  | 13 552 | 12 094 |  |  |  |  |
| Other health care costs**‡** |  |  | 4 078 | 1 800 |  |  |  |  |
| Sub-total |  |  | 17 630 | 13 490 |  |  |  |  |
| B. Direct healthcare costs (annual) | |  |  |  |  |  |  |  |
| Medication cost | 7329 | 10333 | 6973 | 10255 | 5661 | 4208 | 5888 | 4781 |
| GP visits | 2599 | 1692 | 1485 | 1568 | 2631 | 1950 | 2546 | 1702 |
| Other (private) doctors | 3198 | 7170 | 1230 | 2106 | 2999 | 6914 | 2905 | 6074 |
| Psychologists | - | - | 1313 | 5221 | 3214 | 10768 | 6000 | 16248 |
| Physiotherapists | 1446 | 3080 | 2095 | 3864 | 2124 | 4410 | 2562 | 5170 |
| Hospital stays | 7073 | 28420 | - | - | 3233 | 17110 | 4850 | 25665 |
| CAM visits | 1635 | 613 | 770 | 289 | 1319 | 495 | 1539 | 577 |
| Direct travel costs | 2297 | 2149 | 3848 | 1992 | 2532 | 1697 | 2876 | 2972 |
| Total direct cost | 25 577 | 31 356 | 35 344 | 12 582 | 23 714 | 22 374 | 29 166 | 31 957 |
| C. Indirect health care costs (annual) | |  |  |  |  |  |  |  |
| GP visits | 4837 | 3149 | 2764 | 2919 | 4897 | 3629 | 4739 | 3168 |
| Other (private) doctors | 2695 | 6043 | 1037 | 1775 | 2527 | 5826 | 2448 | 5119 |
| Psychologists | - | - | 484 | 1924 | 1185 | 3969 | 2211 | 5988 |
| Physiotherapists | 3386 | 7215 | 4907 | 9052 | 4976 | 10331 | 6002 | 12110 |
| Hospital stays | 1296 | 5206 | - | - | 592 | 3134 | 889 | 4702 |
| CAM visits | 4837 | 6464 | 2972 | 6117 | 3791 | 6365 | 4344 | 6269 |
| Sub-total | 17 051 | 15 798 | 12 164 | 11 607 | 17 968 | 14 830 | 20 633 | 22 263 |
| D. Cost of productivity loss* | 8565 | 29540 | 2244 | 11736 | 14868 | 48605 | 39420 | 110983 |
| E. Total indirect cost (C+D) | 25617 | 35 622 | 14407 | 16 506 | 32836 | 50 817 | 60053 | 117 764 |
| F. Total health care costs (A+B+C) | 42 628 | 35 111 | 47 506 | 21 795 | 41 682 | 26 843 | 49 799 | 38947 |
| G. Societal cost (D+F) | 51193 | 65 060 | 49750 | 61 539 | 56550 | 55 525 | 89219 | 132 607 |

*SD:* Standard deviation*; MUPS* medically unexplained physical symptoms; *GP:* general practitioner; *CAM:* complementary and alternative medicine. **‡**Include direct travel costs and indirect health care costs
