# Supplementary material for: Is amalgam removal in patients with medically unexplained physical symptoms cost-effective? A prospective cohort and decision modelling study in Norway
Source: PLoS One. 2022 Apr 29;17(4):e0267236. doi: 10.1371/journal.pone.0267236 (PMC9053791; doi:10.1371/journal.pone.0267236)
Supplement: Attachment — Submitted filename: Response_to_Reviewers_Comments18.03.22.docx [file pone.0267236.s002.docx]

**Re:** ***Response to editor’s and reviewers’ comments (PONE-D-22-00123)***

Dear Editor(s),

We would like to thank you for the opportunity to submit a revised copy of our manuscript entitled “Is amalgam removal in patients with medically unexplained physical symptoms cost-effective? A prospective cohort and decision modelling study in Norway”*.*

We would like to thank the editor(s) and reviewers for the positive feedback and helpful comments, which we believe have substantially improved our manuscript. Below is a detailed point-by-point response to the comments and suggestions raised in the review with our responses in ***bold italics***. The revision has been developed in consultation with all coauthors, and each author has approved the final form of this revision.

Thank you very much for your consideration of our manuscript.

**Point-by-point responses to comments:**

**(A) Journal Requirements:**

When submitting your revision, we need you to address these additional requirements.

1. Please ensure that your manuscript meets PLOS ONE's style requirements, including those for file naming. The PLOS ONE style templates can be found at 
<https://journals.plos.org/plosone/s/file?id=wjVg/PLOSOne_formatting_sample_main_body.pdf> and <https://journals.plos.org/plosone/s/file?id=ba62/PLOSOne_formatting_sample_title_authors_affiliations.pdf>

***Thank for reminding us. We have now considered all journal requirements.***

2. We note that the grant information you provided in the ***‘***Funding Information’ and ‘Financial Disclosure’ sections do not match.

***Thank for spotting this problem. It has now been revised as:***

**“Funding:** The project was funded by Norwegian Ministry of Health and Care Services (Helse- og Omsorgsdepartementet) via the Norwegian Directorate of Health. The funder had no role in study design, data collection and analysis, decision to publish, or preparation of the manuscript.”

When you resubmit, please ensure that you provide the correct grant numbers for the awards you received for your study in the ‘Funding Information’ section.

***This project has no grant number or reference number. This has been reported when we submitted the manuscript.***

3. In your Data Availability statement, you have not specified where the minimal data set underlying the results described in your manuscript can be found. PLOS defines a study's minimal data set as the underlying data used to reach the conclusions drawn in the manuscript and any additional data required to replicate the reported study findings in their entirety. All PLOS journals require that the minimal data set be made fully available. For more information about our data policy, please see <http://journals.plos.org/plosone/s/data-availability>.

Upon re-submitting your revised manuscript, please upload your study’s minimal underlying data set as either Supporting Information files or to a stable, public repository and include the relevant URLs, DOIs, or accession numbers within your revised cover letter. For a list of acceptable repositories, please see <http://journals.plos.org/plosone/s/data-availability#loc-recommended-repositories>. Any potentially identifying patient information must be fully anonymized.

Important: If there are ethical or legal restrictions to sharing your data publicly, please explain these restrictions in detail. Please see our guidelines for more information on what we consider unacceptable restrictions to publicly sharing data: <http://journals.plos.org/plosone/s/data-availability#loc-unacceptable-data-access-restrictions>. Note that it is not acceptable for the authors to be the sole named individuals responsible for ensuring data access.

We will update your Data Availability statement to reflect the information you provide in your cover letter.

***We included all necessary data related to our analyses reported in the manuscript. However, individual data points were not included due privacy issue, and justification has been provided.***

**Data Availability:** Data contain potentially identifying and sensitive patient information and is not available due to personal data protection regulations. The restrictions are imposed by NORCE's Administrative Support for Research and the Data Protection Office at NORCE. Data requests may be sent to NORCE's Administrative Support for Research ([forskningsstotte@norceresearch.no](mailto:forskningsstotte@norceresearch.no)) with reference to "Project 42564 Prospektiv kohortstudie av helseplager."

4. Please include your full ethics statement in the ‘Methods’ section of your manuscript file. In your statement, please include the full name of the IRB or ethics committee who approved or waived your study, as well as whether or not you obtained informed written or verbal consent. If consent was waived for your study, please include this information in your statement as well.

***Thank for this comment. We have now included the ethics statement in the ‘Methods’ section. It reads as follow:***

***“***The study was approved by the local research ethics committee (REK2012/331) and registered at ClinicalTrials.gov (<https://clinicaltrials.gov/ct2/show/NCT01682278>; NCT01682278). Informed written consent was obtained from all participants in the study.”

**(B) Additional Editor Comments:**

Dear Authors,

After appraising the manuscript and the unanimous decision of the reviewers, I concede a major revision to it.
Please make use of the reviewers comments/
You can mention in the introduction that amalgam is still considered a treatment option. The supporting literature follows:
- Int Endod J. 2017 Oct;50(10):951-966. doi: 10.1111/iej.12723. Epub 2017 Jan 17.
- J Prosthet Dent. 2017 Mar;117(3):345-353.e8. doi: 10.1016/j.prosdent.2016.08.003.

***Thank you for this comment. We have now included a statement including the supporting literature as per your suggestion. (Page 3, end of last par.)***


**(C) Reviewers' comments to the Author**

***Reviewer #1:***

1. The whole manuscript needs English copy editing-

***We have had another round of professional language editing of the manuscript, and hope the English language is now acceptable.***

2. Introduction section. The author needs to highlight more on the main problem of the study. It's a bit ambiguous here, whether the main problem is MUPS or the high demand of amalgam replacement. Please kindly explain

***Thank you for this comment. We have now briefly highlighted the main problem of the study (page 4, paragraph 2)***

3. Method section.
a. The study was performed excellently, with so many details, which is good.  Please add more explanation about the formulating calculation that were used to analyze those acquired information.

***Thank for the comment. We now included further details about the formulating calculations where necessary.***

b. Please explain why amalgam replacement need 12 months to be done. Was that the required time to replace 1 restoration or all the restorations in 1 patient.

***Thank for this comment. Mean length of the treatment period (from acceptance to start treatment to finished treatment) was 253 days (SD 134, range from 29 to 619) for all respondents in Amalgam cohort. The more amalgam surfaces to replace, the longer was the treatment period. The correlation between number of amalgam surfaces to replace and treatment period was significant (p=0.019). We have now added an explanation (Page 5, last paragraph).***

4. The result section. Please move all the tables to the result section. I think it would be better if all the variables appear in the result are tabulated (ex: please write both male and female on table 1)

***We agree with the reviewer. We moved relevant tables to the RESULTS section. We also included all relevant variables in Table 1.***

5. Discussion section. The age of the samples varies between 20-70 years old. Please add an explanation whether there are any confounding factors, like the respondents general health, they might affect the result.

***Thank you for bringing the issue of confounding to our attention. We have now added explanation involving any general health conditions of the respondents (Page 20, last paragraph)***

***Reviewer #2:***

What is standard care that you refer to and compare to?

***Thank you for this comment. The term “standard of care” may be confusing as it implies a uniform or proven practice standard. Thus, for MUPS patients without attribution to amalgam fillings, we replace it by the term “usual care” to describe ‘*de facto’ *clinical care without any value judgment.***

Can you briefly explain how the health care system works in the country in terms of financial burden on individual?

***Norway has universal health and social insurance coverage, known as the National Insurance Scheme (NIS)*. *Health coverage is automatic for all residents and has two main funding sources: the general tax system and household out-of-pocket payments. The share of the latter is quite small, where public sources account for most health expenditures in Norway, at 85 percent. The establishment of universal coverage has a long history in Norway, and beyond the scope of this manuscript. However, we agree with the reviewer and included brief explanation (Page 7, first paragraph)***

I think that the interpretation of what WHO said about dental amalgam is different from what WHO said. The danger is mostly from placent and removal and affects dentist and dental staff more thatn the patient. WHO says that there is lack of evidence to danger of dental amlgam,

***The WHO Consensus Statement on Dental Amalgam from 1997 cited by the World Dental Federation at their web pages (***[***https://www.fdiworlddental.org/***](https://www.fdiworlddental.org/)***) is obsolete, and more recent research express that the thresholds for toxicity related to mercury exposures are uncertain. For example, the recent statement issued by US FDA (September 24, 2020) says “Current estimates of continuous, exposure to mercury from dental amalgam and other sources over a lifetime that are likely to be without risks of harmful effects in the general population and greater risk groups, vary considerably (***[***https://www.fda.gov/medical-devices/safety-communications/recommendations-about-use-dental-amalgam-certain-high-risk-populations-fda-safety-communication***](https://www.fda.gov/medical-devices/safety-communications/recommendations-about-use-dental-amalgam-certain-high-risk-populations-fda-safety-communication)***) … these uncertainties present challenges with regard to defining a specific threshold of toxicity for chronic, low-level mercury exposure from dental amalgam and other sources, particularly for sensitive groups.” Yet, we agree with reviewer and, hence we have now revised the sentence (Page 3, para. 1)***

Is there a relationship between number of removed amalgams and improvement?

***There is a positive relationship between number of removed amalgams and improvement, which will be reported elsewhere. However, a positive (but not statistically significant) correlation between number of amalgam surfaces and symptom score has been reported previously (see Weidenhammer et al 2010). The correlation between inorganic mercury in plasma (a biomarker for exposure to dental amalgam) and symptom score was significant (see Weidenhammer et al 2010, Figure 3).***

***Reference:***

***Weidenhammer W, Bornschein S, Zilker T, Eyer F, Melchart D, Hausteiner C. Predictors of treatment outcomes after removal of amalgam fillings: associations between subjective symptoms, psychometric variables and mercury levels. Community Dent Oral Epidemiol. 2010;38:180-9.***

TABLE 1 does not include male information under gender

***Thank you for spotting this issue. We have now revised.***

Did you perform a power calculation to determine sample size?

# *Thank for the comment. However, issues related to power calculation was reported elsewhere (Björkman et al., 2020), entitled “Removal of dental amalgam restorations in patients with health complaints attributed to amalgam: A prospective cohort study.”* [*https://pubmed.ncbi.nlm.nih.gov/32810306/*](https://pubmed.ncbi.nlm.nih.gov/32810306/) *.* We have now briefly indicated this in the manuscript (Page 5; Para. 1).

What is the average number of amalgam fillings per patient in the amalgam group?

***Mean number of amalgam surfaces was 20.3 (SD 10.9, range from 5 to 59). This has been reported on page 5, par. 1.***

And how is that corelated with study outcomes?

***Number of amalgam surfaces at baseline was not a statistically significant predictor of change. An increase by 10 amalgam surfaces increased odds ratio only slightly (1.245; 95% CI 0.635 to 2.441; p=0.524, n=32) to have a change score of the General Health Complaints index (GHC-index) above the median value of 10.95.***

***Patients with higher concentration of inorganic mercury in serum at baseline had higher odds ratio to have a change score of the GHC index above the median value of 10.95. An increase of 0.5 µg Hg/L increased odds ratio by 2.161 (95% CI 0.903 to 5.174; n=32) (Björkman et al; manuscript in preparation).***

What is the average age of amalgam in each patient? What are the conditions of amalgams prior to removal? Looking at failure rate of fillings/crowns that replaced those amalgam would give a complete picture or cost-effectiveness of removal. I understand that the data is not available now but could be considered as a limitation.

***Thank for raising this important point. Since the annual failure rate for the restorations placed in this study is not available, we have now acknowledged the limitations in the discussion section (Page 21, para. 1).***

***Reviewer #3:***

All is appropriately done. However, page 3, there is need to correct the ***references 5*** and ***6*** in line with the journal requirements

Well written paper. Correct references 5 and 6 on page 3 to conform with journal requirements

***Thank for your positive comments and spotting the errors. These references are now corrected.***

***Reviewer #4:***

This study titled ‘’ Is amalgam removal in patients with medically unexplained physical symptoms cost effective? A prThis study titled ‘’ Is amalgam removal in patients with medically unexplained physical symptoms cost effective? A prospective cohort study in Norway” assessed the cost-effectiveness of the removal of amalgam restorations in patients with medically unexplained physical symptoms (MUPS) attributed to amalgam fillings compared to standard care, based on a prospective cohort study in Norway. The study provides understanding about the costs and health outcomes associated with the removal of amalgam restorations in patients who attribute health complaints to dental amalgam restorations. The study emphasized that the available clinical and cost data indicate that amalgam fillings removal in patients who attribute health complaints to dental amalgam restorations is a highly cost-effective intervention.
It also highlighted that the estimated incremental cost per QALY decreased with increasing time horizon, and amalgam removal was found to be cost saving over both 5 and 10 years. The study is indeed interesting, and it tackles an important topic for both dentists and policy makers. Overall, the paper was very well written, but it needed some revisions to improve the clarity. In this context, some clarifications are needed.

Methods
1. The reason why you recruited a healthy group (Healthy cohort) in your study is not clear, your analysis and interpretation of data focused primarily on the amalgam cohort and the MUPS cohort. Accordingly, there is no need to mention the third group that was not included in your analysis.

***We generally agree with the reviewer that the “Healthy Cohort” was not included in our analysis in this manuscript. However, this group was part of the mother project and worth mentioning for the purpose of transparency. We have written (page 4) “This analysis is based on the Amalgam cohort (intervention group) and MUPS cohort (comparator group) and included participants who responded to both the baseline and follow-up questionnaires.” We have now included additional information directly related to the ‘healthy cohort’: “Data for the healthy cohort was not utilized, as this group is not considered eligible for the intervention” (page 5, para. 2).***

2. You mentioned that the inclusion and exclusion criteria for the subjects are published in another article (15). However, some of these criteria must be included in your study e.g whether the MUPS group had amalgam fillings or not? , and the minimal number of fillings in the amalgam cohort.

***Thank for the comments. The MUPS cohort was not examined by a dentist, and thus it was not an inclusion criterion for the MUPS cohort to have amalgam fillings. However, inorganic mercury in serum (which correlates with number of amalgam surfaces) was not significantly different from the amalgam cohort (48.1 % in the MUPS cohort had values ≥ 0.2 µg/L, while 50 % in the Amalgam cohort had values ≥ 0.2 µg/L, p=0.887). Thus, there is no evidence that the exposure was different in the two cohorts. Yet, we agreed with the reviewer, and key relevant criteria have now been included (page 5, para. 1).***

3. The sample size calculation was not mentioned and accordingly it is not clear why you chose to include 60 patients in your study.

***Thank for the comment. Issues related to power calculation was reported elsewhere (Björkman et al., 2020), entitled “Removal of dental amalgam restorations in patients with health complaints attributed to amalgam: A prospective cohort study.”*** [*https://pubmed.ncbi.nlm.nih.gov/32810306/*](https://pubmed.ncbi.nlm.nih.gov/32810306/)

***Moreover, studies strongly warn not to perform a post-hoc power analysis, because post hoc power analysis identifies population-level parameters with sample-specific statistics, makes no conceptual sense and can be misleading (Zhang et al., 2019; Lakens, 2022). Although power analysis is a crucial for planning clinical research studies, it is both conceptually flawed and analytically misleading when used to indicate power for outcomes already observed (like the present study). Despite this challenge, a previous study showed a mean difference in a general health complaint of 10.0 between groups (treatment and reference groups) with a common within standard deviation of 10.0 produced a sample size of 20 in each group with a power of 87% (a two-tailed test at α =5%) Sjursen et al., 2011). A sample size calculation was performed during planning of the mother project on which the present study is based. The calculation was reported elsewhere (Björkman et al., 2020).***

***References:***

1. ***Zhang et al. (2019). Post hoc power analysis: is it an informative and meaningful analysis? General Psychiatry;32:e100069. doi:10.1136/ gpsych-2019-100069.***
2. ***D. Lakens (2022). A relevant blog post on: “What to do if Your Editor Asks for Post-hoc Power?” available at*** [***http://daniellakens.blogspot.com/2014/12/observed-power-and-what-to-do-if-your.html***](http://daniellakens.blogspot.com/2014/12/observed-power-and-what-to-do-if-your.html)***, which is also included in a paper “Sample Size Justification. Collabra: Psychology»available at*** [***PsyArXiv***](https://psyarxiv.com/9d3yf/) ***(https://psyarxiv.com/9d3yf/)***
3. ***Sjursen et al. (2011). Changes in health complaints after removal of amalgam Fillings. Journal of Oral Rehabilitation; 38; 835–848.***
4. ***Björkman et al. (2020). Removal of dental amalgam restorations in patients with health complaints attributed to amalgam: A prospective cohort study. J Oral Rehabil.;47:1422–1434. DOI: 10.1111/joor.13080.***
